# Migraine or any headaches and white matter hyperintensities and their progression in women and men

**DOI:** 10.1186/s10194-024-01782-7

**Published:** 2024-05-15

**Authors:** Sara Helena Schramm, I. Tenhagen, M. Jokisch, J. Gronewold, S. Moebus, S. Caspers, Z. Katsarava, R. Erbel, A. Stang, B. Schmidt

**Affiliations:** 1 Institute for Medical Informatics, Biometry and Epidemiology (IMIBE), University Hospital Essen, Hufelandstr. 55, D-45122 Essen, Germany; 2 Department of Neurology, University Hospital Essen, University Duisburg-Essen, Essen, Germany; 3 Institute for Urban Public Health, University Hospital Essen, University Duisburg-Essen, Essen, Germany; 4 Institute for Anatomy I, Medical Faculty & University Hospital Düsseldorf, Heinrich Heine University Düsseldorf, Düsseldorf, Germany; 5 Institute of Neuroscience and Medicine (INM-1), Research Centre Jülich, Jülich, Germany; 6 Department of Neurology, Christian Hospital Unna, Holbeinstr. 10, 59423 Unna, Germany; 7 EVEX Medical Corporation, 40 Vazha-Pshavela Avenue, Tbilisi, 0177 Georgia; 8 Department of Epidemiology, School of Public Health, Boston University, Boston, USA

**Keywords:** 1000BRAINS, Migraine, Headache, Aura, White matter lesions, White matter hyperintensities, MRI

## Abstract

**Background:**

Cross-sectional and longitudinal studies have been conducted to investigate the association between migraine and any headache and white matter hyperintensities (WMH). However, studies are inconsistent regarding the strength of the association and its clinical significance. The aim of our study was to investigate the association between headache and its subtypes (migraine with aura (MigA+), migraine without aura (MigA-), non-migraine headache (nonMigHA)) and WMH and its course in the population-based 1000BRAINS study using state-of-the-art imaging techniques and migraine classification according to modified international classification of headache disorders.

**Methods:**

Data from 1062 participants (45% women, 60.9 ± 13.0 years) with ever or never headache (neverHA) and complete quantitative (WMH volume) and qualitative (Fazekas classification) WMH data at first imaging and after 3.7 ± 0.7 years (393 participants) were analyzed. The sex-specific association between headache and its subtypes and WMH volume and its change was evaluated by linear regression, between headache and its subtypes and Fazekas score high vs. low (2–3 vs. 0–1) by log-binomial regression, adjusted for confounders.

**Results:**

The lifetime prevalence of headache was 77.5% (10.5% MigA+, 26.9% MigA-, 40.1% nonMigHA). The median WMH volume was 4005 (IQR: 2454–6880) mm3 in women and 4812 (2842–8445) mm3 in men. Women with any headaches (all headache types combined) had a 1.23 [1.04; 1.45]-fold higher WMH volume than women who reported never having had a headache. There was no indication of higher Fazekas grading or more WMH progression in women with migraine or any headaches. Men with migraine or any headaches did not have more WMH or WMH progression compared to men without migraine or men who never had headache.

**Conclusions:**

Our study demonstrated no increased occurrence or progression of WMH in participants with mgiraine. But, our results provide some evidence of greater WMH volume in women with headache of any type including migraine. The underlying pathomechanisms and the reasons why this was not shown in men are unclear and require further research.

**Supplementary Information:**

The online version contains supplementary material available at 10.1186/s10194-024-01782-7.

## Introduction

Migraine represents a major challenge for the health care system and society due to the high prevalence, the strong negative impact on the lives of those affected [1, 2] and the high socioeconomic costs it causes [3, 4]. Not only the disease itself, but also relevant comorbidities such as psychiatric disorders or chronic pain syndromes contribute to the enormous disease burden [5]. Associations with other serious diseases such as the increased risk of stroke, which has been demonstrated in several studies in patients with migraine with aura (MigA+), are also increasingly coming into focus and should be included in therapy and prevention concepts in migraine patients [6, 7]. Several studies using magnetic resonance imaging (MRI) have been performed to investigate the association between migraine and white matter hyperintensities of presumed vascular origin (WMH) as an indicator for cerebral small vessel disease and a possible risk factor for stroke and cognitive impairment [8, 9]. But, the studies are inconsistent regarding the strength of the association and its clinical significance [10, 11]. Also follow-up studies focusing on the changes of WMH in individuals with migraine [11–14] show mixed results. In fact, some studies have described an association with headaches in general and the severity of headaches [11, 15].

The aim of our study was to investigate the association between MigA + and migraine without aura (MigA-) or any headaches and WMH and their progression in the large population-based sample of the longitudinally designed 1000BRAINS study with state-of-the-art imaging and migraine classification according to modified international classification of headache disorders [16, 17]. Because prevalence of migraine and WMH differs between women and men, we performed sex-stratified analyses.

## Methods

### Study population

**Fig. 1:**
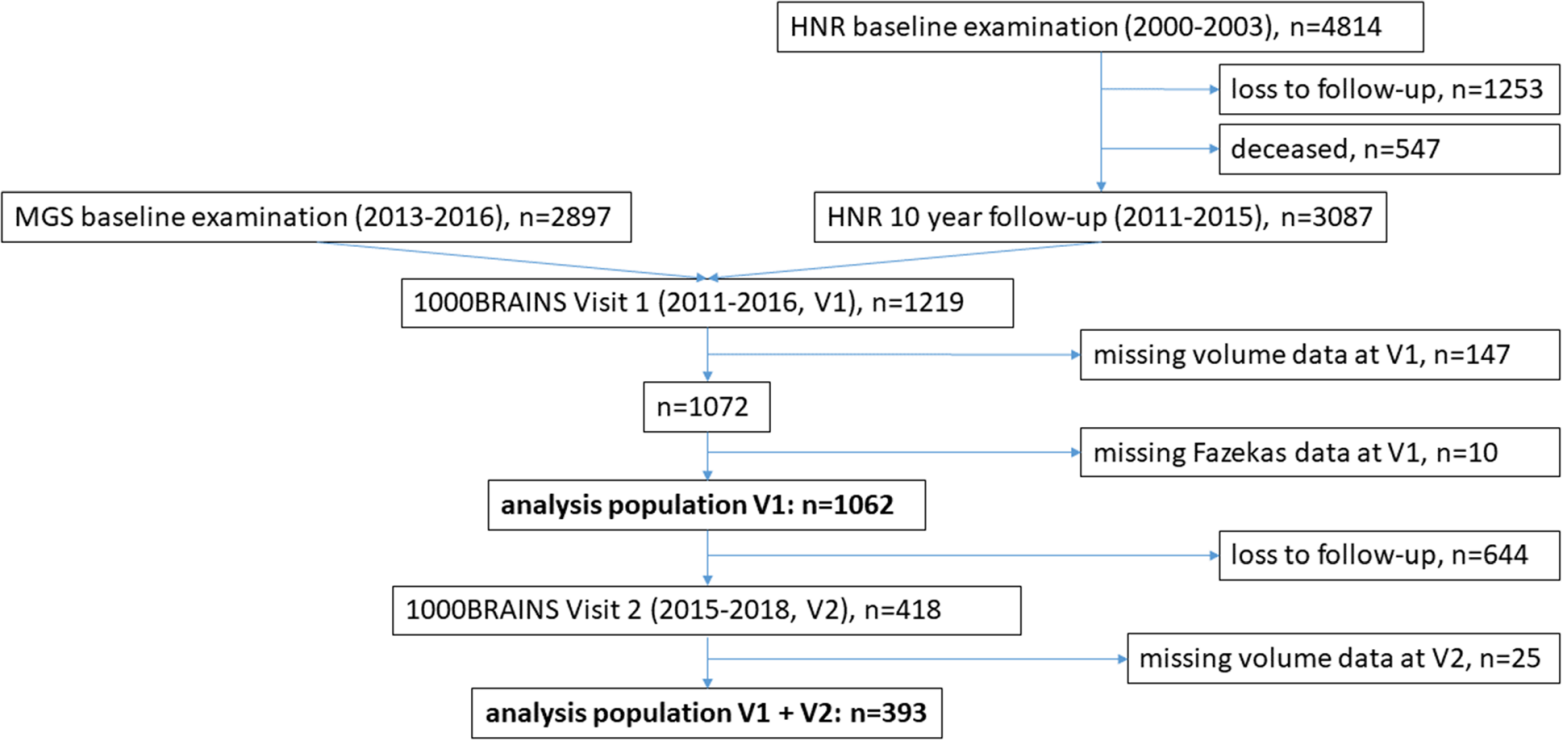
Flowchart of study population. *HNR, Heinz Nixdorf Recall; MGS, Heinz Nixdorf Multigeneration Study; V1, visit 1; V2, visit2*

Data from the 1000BRAINS study were analyzed. The 1000BRAINS study is a longitudinal cohort study at the Institute of Neuroscience and Medicine, Research Centre Jülich, Germany, designed to study variability in brain structure, function, and connectivity during ageing. Details about the study can be found in Caspers, et al. [18]. Briefly, the 1000BRAINS sample is drawn from the 10-year follow-up of the Heinz Nixdorf Recall (HNR) study [19] and the Heinz Nixdorf Multigeneration Study (MGS) [20]. The ongoing population-based prospective HNR study collects data on health, social and environmental risk factors and cardiovascular outcomes in the Ruhr metropolitan region. The study population was composed of random samples from the population registers of the cities of Bochum, Essen, and Mülheim/Ruhr. Between 2000 and 2003, 4814 participants (aged 45–75 years, 50.2% women) were included in the baseline survey, with a recruitment rate of 55.8% [21]. The MGS is an extension of the HNR study. From 2013 to 2016, the partners and adult children of the HNR participants aged 18–90 years were included in the study for the first time. More details on the HNR study and the MGS and how the 1000BRAINS study emerged from these two studies can be found in the supplementary material (SM) 1 (SM1). The 1000BRAINS study was approved by the ethical committee of the University Duisburg-Essen, Germany. All participants gave written informed consent. *N* = 1219 1000BRAINS participants received MRI at visit 1 (2011–2016, V1), of those *n* = 147 with missing WMH-volume data and *n* = 10 with missing Fazekas data were excluded. The final analysis population at V1 consisted of *n* = 1062 participants. Of those, *n* = 418 participants had an MRI follow-up examination on average 3.7 ± 0.7 years later (visit 2 (V2)). *N* = 25 with missing WMH-volume data at V2 were excluded. The final analysis population at V2 consisted of *n* = 393 participants (Fig. 1).

### Measurements

Computer-assisted personal interviews, clinical examinations, laboratory tests, and MRI were performed according to standard protocols. Questionnaires addressed behavioral risk factors, complete medical history, and sociodemographic characteristics [18, 19].

### Exposure variable headache status

HNR participants provided headache information at the 10-year follow-up and MGS participants at baseline, few weeks before MRI. The interview was computer-assisted by trained study personnel. The diagnostic headache questions were based on modified international classification of headache disorders, 2nd edition (ICHD-II) of the International Headache Society (IHS) [16, 17] asking “Have you ever had a headache in your life?” and “If you had this headache, did you have symptom xy?“. The headache questions were designed by a headache specialist and introduced at the time of the second follow-up of the HNR study and at the baseline survey of the MGS. More details are in the supplement of [17]. Headache status is defined in Table 1.

**Table 1:** Definition of headache status according to the modified* criteria of the International Headache Society (IHS) [16, 17]

headache	migraine	definite MigA+	definite MigA + according to the modified criteria of the IHS and one of the following accompanying symptoms immediately before headache: visual impairments (like flickering, streaks, lines, scotoma, zigzag figures), spreading sensoric paresthesia or numbness, motoric dysfunction or disorders of speech or language
		probable MigA+	headache or probable MigA + according to the modified criteria of the IHS and one of the following accompanying symptoms immediately before headache: visual impairments (like flickering, streaks, lines, scotoma, zigzag figures), spreading sensoric paresthesia or numbness, motoric dysfunction or disorders of speech or language
		definitive MigA-	according to the modified criteria of the IHS
		probable MigA-	according to the modified criteria of the IHS
	non-migraine	nonMigHA	any headache not fulfilling the criteria for MigA + and MigA-
neverHA		neverHA	remaining participants who stated they had never had a headache in their lives

MigA+, migraine with aura; MigA-, migraine without aura; nonMigHA, non-migraine headache; neverHA, never headache; IHS, International Headache Society

*The questionnaire was a modified version of the validated questionnaire of the German Headache Consortium study [17, 22]. The original questionnaire was developed and validated for headaches and migraine symptoms within the last 12 months, we asked for headache and migraine symptoms that had ever occurred in the lifetime

If the text does not explicitly mention definite or possible MigA + or MigA-, the participants with definite or possible migraine (MigA + or MigA-, respectively) were pooled.

### White matter hyperintensities of presumed vascular origin (WMH)

MRI at V1 and V2 was carried out on the same 3 Tesla MR scanner (Tim-TRIO, Siemens Medical Systems, Erlangen, Germany) using a 32-channel head coil. The T2-weighted structural brain images [fluid-attenuated inversion recovery (FLAIR)) scanned with: repetition time (TR) = 9s, echo time (TE) = 100ms, FoV = 220 × 220mm^2^, flip angle = 150°, voxel resolution = 0.9 × 0.9 × 4mm^3^, 25 slices] were used for quantitative and qualitative WMH determination. The quantitative WMH-volume data for V1 and V2 in mm^3^ was calculated with the BIANCA (Brain Intensity AbNormality Classification Algorithm) software [23]. A qualitative evaluation was done by two independent raters, who evaluated the location and extent of WMH according to the qualitative rating scale of Fazekas [24, 25], also using the FLAIR sequences. A moderate interrater agreement was achieved (Cohens kappa = 0.56 for deep and 0.58 for periventricular WMH). In case of interrater disagreement, the raters met to reach a consent [26]. Deep and periventricular WMH were graded as shown in Table 2.

**Table 2:** Fazekas classification according to Fazekas et al. 1987 [25]

Grad	deep WMH	periventricular WMH
0	absence	absence
1	punctate foci	‘caps’ or pencil-thin lining
2	beginning confluence of foci	smooth ‘halo’
3	large confluent areas	irregular periventricular hyperintensities extending into the DWM

WMH, white matter hyperintensities

**Fig. 2:**
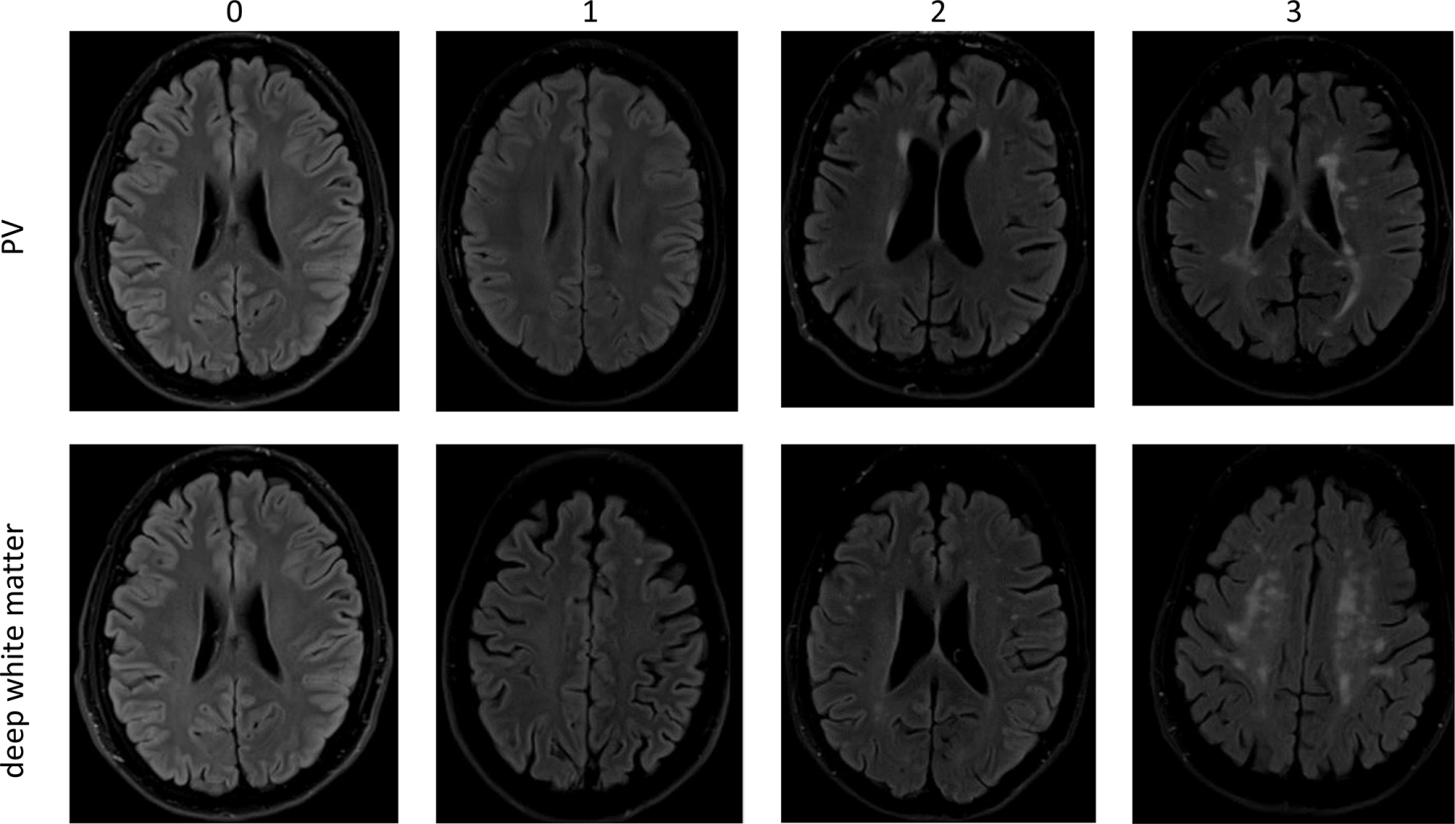
Transverse slice MRI images in FLAIR sequence (fluid attenuated inversion recovery) with examples of periventricular (PV) and deep white matter lesions (modified image from [27]) according to Fazekas et al. [25]

Raters were blinded to all other participants’ data and risk factors at the time of assessment. Figure 2 shows transverse slice examples in FLAIR sequence from our study corresponding to different Fazekas scores.

Because in older populations it is normal to have a small amount of WMH we converted the 4-level Fazekas score into a binary variable by combining grades 0 and 1 (low score) and grades 2 and 3 (high score). The difference of the Fazekas scores, V2 minus V1, can take values between − 3 and + 3. Negative values correspond to an improvement and positive values to a progression of WMH. The value 0 corresponds to no change. For further analysis, the categories for improvement and no change were combined to “no progression”.

Outcome variables for the WMH at V1 were (1) WMH volume in mm^3^ and (2) low (0–1) vs. high (2–3) Fazekas score. Outcome variables for WMH change after 3.7 years were (3) absolute change of the WMH volume in mm^3^ (WMH volume difference = WMH volume at V2 - WMH volume at V1, divided by the time from V1 to V2 in years times 3.7 years), (4) relative change of the WMH volume in % (relative WMH volume change = (WMH volume at V2 - WMH volume at V1) / (WMH volume at V1), divided by the time from V1 to V2 in years times 3.7 years), and (5) change of Fazekas score (no progression vs. progression).

### Confounder variables and covariates

Included confounder variables identified by directed acyclic graph (DAG) (SM2) at the HNR 10-year follow-up and MGS baseline were current smoking (history of cigarette smoking during the past year), past smoking (quitting smoking more than a year ago), otherwise ‘never smoking’; body mass index (BMI) in kg/m² (calculated according to the equation ‘measured body weight/ measured height²); sports (‘yes’ when practiced in the last 4 weeks before the interview, otherwise ‘no’); years of education (according to the International Standard Classification of Education (ISCED-97) as the total number of years of formal education, which includes both schooling and vocational training); age in years, and sex. Further covariates to characterize the study population were blood pressure (measured using an oscillometric method (Omron; Netherlands), taking the mean value of the second and third of three measurements at least 2 min apart), diabetes mellitus (when fasting glucose exceeded ≥ 126 mg/dl or participants reported use of insulin or oral hypoglycaemic agents), total, low-density lipoprotein (LDL), and high-density lipoprotein (HDL) blood cholesterol levels in mg/dl (determined by standardized enzymatic methods using the ADVIA 1650 system, Siemens Healthcare Diagnostik, Eschborn, Germany).

### Statistical analysis

The cross-sectional analysis was based on the data of *n* = 1062 participants with complete WMH-volume data and Fazekas classification at V1 and known headache status. Investigation of the progression of WMH was based on *n* = 393 participants with complete WMH-volume data and Fazekas classification at V1 and V2 and known headache status. Descriptive statistics stratified by headache status and sex were performed (mean ± standard deviation (SD) for continuous variables; median and quartiles (Q1, Q3) for WMH-volume data; absolute frequencies and percentage for dichotomous and categorical variables). Cross sectional, we compared (1) definite and probable MigA + and MigA- vs. participants without migraine and (2) definite and probable MigA + and MigA-, and nonMigHA vs. neverHA, stratified by sex. The association between headache status and WMH volume at V1 was evaluated by log-transforming (natural logarithm) the skewed distributed WMH-volume variable and by using linear regression, adjusted for confounding (model 1: adjusted for age; model 2: as model 1 + smoking status (never/past/current), BMI, sport (yes/no) and years of education (according to the DAG [28] used to determine the minimal sufficient adjustment set, see Figure SM2); model 3: as model 2 + diabetes mellitus, systolic blood pressure, and cholesterol which are known WMH risk factors. Exponents of beta (β) - estimates and corresponding 95%-confidence intervals [95%CI] were calculated. The associations between headache status and deep and periventricular Fazekas score (high vs. low) were evaluated by multiple log-binomial regression using the same adjustment sets. Prevalence ratios (PR) and 95%CI were calculated. The associations between headache status and the absolute and relative change in % of the WMH volume were also evaluated by linear regression.

## Results

### Descriptive statistics of WMH at V1

The characteristics of the study population at V1 (*n* = 1062, 45% women, age range: 18–84 years, mean age: 60.9 ± 13.0 years) are presented in Table 3 (women) and Table 4 (men) stratified by headache status. As expected, the proportions of participants with migraine were higher in women than in men (MigA+: 14.6% vs. 7.2%; MigA-: 35.8% vs. 19.6%). The characteristics of study participants with definite and probable MigA + and definite and probable MigA-, respectively, were similar (gray font color). As expected, main aura symptoms were visual disturbance (97.1% of women and 88.1% of men with MigA+), the other aura symptoms were less common. Participants with migraine with and without aura had a lower mean age and participants in the neverHA category had the highest mean age, in both sexes. Only *n* = 4 women ≤ 55 years reported neverHA (SM3). The overall proportion of active smokers was the same in women and men (13.3% vs. 13.1%). Mean BMI amounted to around 27 kg/m² (overweight) in women and men. Women reported sport somewhat more frequently than men (70.0% vs. 65.3%). On average, women had completed fewer years of education (14.2 ± 2.3 years vs. 15.5 ± 2.2 years), had lower systolic blood pressure (123.2 ± 16.8 mmHg vs. 131.7 ± 16.4 mmHg), less often diabetes mellitus (10.0% vs. 16.0%), and higher total, LDL, and HDL cholesterol levels than men. As expected, higher WMH volumes were uncommon in younger participants and volume appeared to increase exponentially with age (Figure SM4). Women had a median WMH volume of 4005 mm^3^ and men of 4812 mm^3^. Within women, women with nonMigHA had the highest median WMH volume of 4125 mm^3^. Within men, men with neverHA had the highest median WMH volume of 5833 mm^3^. There was no evidence for higher Fazekas scores at V1 in participants with migraine (Tables 3 and 4 and SM5).

**Table 3:** Characteristics of the female study population at **visit 1**, stratified by headache status; *n* = 480, n(%), mean ± SD, median (Q1; Q3)

	Definitive MigA+*	Probable MigA+*	All MigA+**	Definitive MigA-*	Probable MigA-*	All MigA−**	NonMigHA	NeverHA	Total
n(%)	*40 (8.3)*	*30 (6.3)*	70 (14.6)	*78 (16.2)*	*94 (19.6)*	172 (35.8)	169 (35.2)	69 (14.4)	480 (100)
visual disturbances***	*38 (95.0)*	*30 (100)*	68 (97.1)						
tingling/deafness***	*12 (30.0)*	*3 (10.0)*	15 (21.4)						
weakness in arm/leg***	*3 (7.5)*	*0*	3 (4.3)						
speech disorders***	*6 (15.0)*	*3 (10.0)*	9 (12.9)						
age [years]	*58.3 ± 11.0*	*60.2 ± 12.8*	59.1 ± 11.7	*59.0 ± 13.2*	*57.4 ± 14.1*	58.1 ± 13.7	61.8 ± 10.9	66.5 ± 8.2	60.8 ± 12.1
smoking never past current	*21 (52.5)*	*18 (60.0)*	39 (55.7)	*40 (51.3)*	*54 (57.4)*	94 (54.7)	92 (54.4)	42 (60.9)	267 (55.6)
	*15 (37.5)*	*6 (20.0)*	21 (30.0)	*29 (37.2)*	*29 (30.9)*	58 (33.7)	50 (29.6)	20 (29.0)	149 (31.0)
	*4 (10.0)*	*6 (20.0)*	10 (14.3)	*9 (11.5)*	*11 (11.7)*	20 (11.6)	27 (16.0)	7 (10.1)	64 (13.3)
BMI [kg/m²]	*25.9 ± 4.6*	*26.7 ± 4.0*	26.3 ± 4.3	*27.6 ± 5.1*	*27.3 ± 5.5*	27.4 ± 5.3	27.2 ± 5.0	27.6 ± 4.7	27.2 ± 5.0
missing					*1*	1			1
sport yes no	*28 (70.0)*	*21 (70.0)*	49 (70.0)	*53 (67.9)*	*64 (68.1)*	117 (68.0)	118 (69.8)	52 (75.4)	336 (70.0)
	*12 (30.0)*	*9 (30.0)*	21 (30.0)	*25 (32.1)*	*30 (31.9)*	55 (32.0)	51 (30.2)	17 (24.6)	144 (30.0)
education [years]	*14.4 ± 2.3*	*14.6 ± 2.4*	14.5 ± 2.3	*14.5 ± 2.2*	*14.3 ± 2.4*	14.4 ± 2.3	14.2 ± 2.3	13.7 ± 2.2	14.2 ± 2.3
missing	*1*		1		*2*	2			3
systolic RR [mmHg]	*123.7 ± 14.6*	*118.1 ± 15.8*	121.3 ± 15.7	*122.2 ± 17.4*	*124.4 ± 16.8*	123.4 ± 17.1	123.0 ± 17.4	124.7 ± 15.9	123.2 ± 16.8
diabetes mellitus yes no	*3 (7.5)*	*1 (3.3)*	4 (5.7)	*9 (11.5)*	*12 (12.8)*	21 (12.2)	15 (8.9)	8 (11.6)	48 (10.0)
	*37 (92.5)*	*29 (96.7)*	66 (94.3)	*69 (88.5)*	*82 (87.2)*	151 (87.8)	154 (91.1)	61 (88.4)	432 (90.0)
cholesterol [mg/dl]	*225.2 ± 44.5*	*206.8 ± 34.4*	217.4 ± 41.3	*229.7 ± 38.2*	*220.3 ± 40.6*	224.6 ± 39.7	222.0 ± 38.9	227.9 ± 40.7	223.1 ± 39.8
missing		*1*	1	*2*	*2*	4	1		6
LDL [mg/dl]	*129.5 ± 36.7*	*124.1 ± 29.3*	127.2 ± 33.6	*137.8 ± 34.4*	*128.3 ± 35.4*	132.6 ± 35.2	128.3 ± 32.2	133.3 ± 36.7	130.4 ± 34.1
missing		*1*	1	*2*	*1*	4			5
HDL [mg/dl]	*72.4 ± 14.3*	*65.4 ± 16.5*	69.4 ± 15.5	*71.4 ± 17.5*	*70.9 ± 18.1*	71.1 ± 17.8	71.5 ± 16.6	71.3 ± 16.9	71.0 ± 16.9
missing		*1*	1	*2*	*2*	4			5
WMH vol. V1 [mm^3^] median	*4069*	*4117*	4082	*3758*	*3458*	3625	4125	4078	4005
Q1; Q3	*2022; 7462*	*2116; 8437*	2039; 7688	*2051; 6594*	*2339; 6475*	2072; 6534	2727; 7512	2842; 6091	2454; 6880
Fazekas grade (deep) low (0–1)	*26 (65.0)*	*17 (56.7)*	43 (61.4)	*55 (70.5)*	*64 (68.1)*	119 (69.2)	104 (61.5)	38 (55.1)	304 (63.3)
high (2–3)	*14 (35.0)*	*13 (43.3)*	27 (38.6)	*23 (29.5)*	*30 (31.9)*	53 (30.8)	65 (38.5)	31 (44.9)	176 (36.7)
Fazekas grade (pv) low (0–1)	*23 (57.5)*	*19 (63.3)*	42 (60.0)	*48 (61.5)*	*66 (70.2)*	114 (66.3)	100 (59.2)	42 (60.9)	298 (62.1)
high (2–3)	*17 (42.5)*	*11 (36.7)*	28 (40.0)	*30 (38.5)*	*28 (29.8)*	58 (33.7)	69 (40.8)	27 (39.1)	182 (37.9)

BMI, body mass index; LDL, low-density lipoprotein; HDL, high-density lipoprotein; MigA+, migraine with aura; MigA-, migraine without aura; neverHA, never headaches; nonMigHA, non-migraine headache; pv, periventricular; Q1, lower quartile; Q3, upper quartile; RR, blood pressure; SD, standard deviation; vol., volume; WMH, white matter hyperintensity. *definite and probable migraine with and without aura according to modified ICHD-II classification. **definite and probable MigA + and MigA-, respectively, combined. ***immediately before the headache

**Table 4:** Characteristics of the **male** study population at **visit 1**, stratified by headache status; *n* = 582, n(%), mean ± SD, median (Q1; Q3)

	definitive MigA+*	probable MigA+*	MigA+**	definitive MigA-*	probable MigA-*	MigA−**	nonMigHA	neverHA	total
**n(%)**	*12 (2.1)*	*30 (5.2)*	42 (7.2)	*37 (6.4)*	*77 (13.2)*	114 (19.6)	257 (44.2)	169 (29.0)	582 (100)
visual disturbances***	*11 (91.7)*	*26 (86.7)*	37 (88.1)						
tingling/deafness***	*1 (8.3)*	*4 (13.3)*	5 (11.9)						
weakness in arm/leg***	*2 (16.7)*	*2 (6.7)*	4 (9.5)						
speech disorders***	*0*	*3 (10.0)*	3 (7.1)						
**age [years]**	*56.0 ± 13.6*	*55.8 ± 12.7*	55.9 ± 12.8	*56.9 ± 14.9*	*58.3 ± 12.7*	57.8 ± 14.6	60.1 ± 13.8	65.6 ± 11.7	60.9 ± 13.7
**smoking never** ** past** ** current** missing	*3 (25.0)*	*13 (43.3)*	16 (38.1)	*16 (43.2)*	*28 (36.4)*	44 (38.6)	105 (40.9)	62 (36.7)	227 (39.0)
	*8 (66.7)*	*12 (40.0)*	20 (47.6)	*13 (35.1)*	*41 (53.2)*	54 (47.4)	114 (44.4)	90 (53.3)	278 (47.8)
	*0*	*5 (16.7)*	5 (11.9)	*8 (21.6)*	*8 (10.4)*	16 (14.0)	38 (14.8)	17 (10.1)	76 (13.1)
	*1 (8.3)*		1 (2.4)						1 (0.2)
**BMI [kg/m²]**	*29.4 ± 5.2*	*27.4 ± 4.0*	28.0 ± 4.4	*26.9 ± 3.3*	*28.5 ± 4.8*	28.0 ± 4.4	27.8 ± 3.9	27.7 ± 3.5	27.8 ± 3.9
missing						1	1	1	3
**sport yes** ** no** missing	*7 (58.3)*	*21 (70.0)*	28 (66.7)	*30 (81.1)*	*50 (64.9)*	80 (70.2)	167 (65.0)	105 (62.1)	380 (65.3)
	*4 (33.3)*	*9 (30.0)*	13 (31.0)	*7 (18.9)*	*27 (35.1)*	34 (29.8)	90 (35.0)	64 (37.9)	201 (34.5)
	*1 (8.3)*		1 (2.4)						1 (0.2)
**education [years]**	*14.0 ± 2.3*	*15.9 ± 2.4*	15.4 ± 2.5	*15.9 ± 2.1*	*16.0 ± 2.0*	16.0 ± 2.0	15.5 ± 2.2	15.3 ± 2.2	15.5 ± 2.2
missing						1			1
**systolic RR [mmHg]**	*131.0 ± 7.5*	*127.0 ± 18.7*	128.1 ± 16.4	*128.4 ± 13.6*	*129.9 ± 18.9*	129.4 ± 17.3	131.7 ± 15.2	134.3 ± 17.3	131.7 ± 16.4
missing			1					1	2
**diabetes mellitus yes** ** no**	*3 (25.0)*	*4 (13.3)*	7 (16.7)	*2 (5.4)*	*12 (15.6)*	14 (12.3)	36 (14.0)	36 (21.3)	93 (16.0)
	*9 (75.0)*	*26 (86.7)*	35 (83.3)	*35 (94.6)*	*65 (84.4)*	100 (87.7)	221 (86)	133 (78.7)	489 (84.0)
**cholesterol [mg/dl]**	*206.2 ± 49.1*	*202.0 ± 50.4*	203.2 ± 49.4	*210.5 ± 41.5*	*204.0 ± 36.0*	206.2 ± 37.8	204.8 ± 38.6	209.3 ± 34.6	206.3 ± 38.2
missing			1			2	2		5
**LDL [mg/dl]**	*114.7 ± 35.2*	*123.3 ± 32.4*	120.8 ± 33.0	*133.6 ± 35.5*	*126.0 ± 35.4*	128.5 ± 35.5	127.9 ± 33.5	127.7 ± 32.8	127.5 ± 33.6
missing			1			2	2		5
**HDL [mg/dl]**	*54.6 ± 18.8*	*52.4 ± 14.5*	53.0 ± 15.6	*54.5 ± 11.1*	*51.5 ± 10.6*	52.5 ± 10.8	54.9 ± 13.7	57.4 ± 14.7	55.0 ± 13.7
missing			1			2	2		5
**WMH vol. V1 [mm**^**3**^**]** median Q1; Q3	*4184* *3199; 6980*	*3189* *1959; 5807*	3762 2527; 5807	*4105* *2312; 5192*	*4662* *2573; 7738*	4349 2412; 6736	4731 2830; 7742	5833 3671; 10,592	4812 2842; 8445
**Fazekas grade (deep)** low (0–1)	*8 (66.7)*	*24 (80.0)*	32 (76.2)	*27 (73.0)*	*53 (68.8)*	80 (70.2)	177 (68.9)	102 (60.4)	391 (67.2)
high (2–3)	*4 (33.3)*	*6 (20.0)*	10 (23.8)	*10 (27.0)*	*24 (31.2)*	34 (29.8)	80 (31.1)	67 (39.6)	191 (32.8)
**Fazekas grade (pv)** low (0–1)	*9 (75.0)*	*21 (70.0)*	30 (71.4)	*7 (73.0)*	*55 (71.4)*	82 (71.9)	152 (59.1)	88 (52.1)	352 (60.5)
high (2–3)	*3 (25.0)*	*9 (30.0)*	12 (28.6)	*20 (27.0)*	*22 (28.6)*	32 (28.1)	105 (40.9)	81 (47.9)	230 (39.5)

BMI, body mass index; LDL, low-density lipoprotein; HDL, high-density lipoprotein; MigA+, migraine with aura; MigA-, migraine without aura; neverHA, never headaches; nonMigHA, non-migraine headache; pv, periventricular; Q1, lower quartile; Q3, upper quartile; RR, blood pressure; SD, standard deviation; vol., volume; WMH, white matter hyperintensity. *definitive and probable migraine with and without aura according to modified ICHD-II classification. **definite and probable MigA + and MigA-, respectively, combined. ***immediately before the headache

### Association between headache status and WMH at V1 (Fig. 3)

**Fig. 3:**
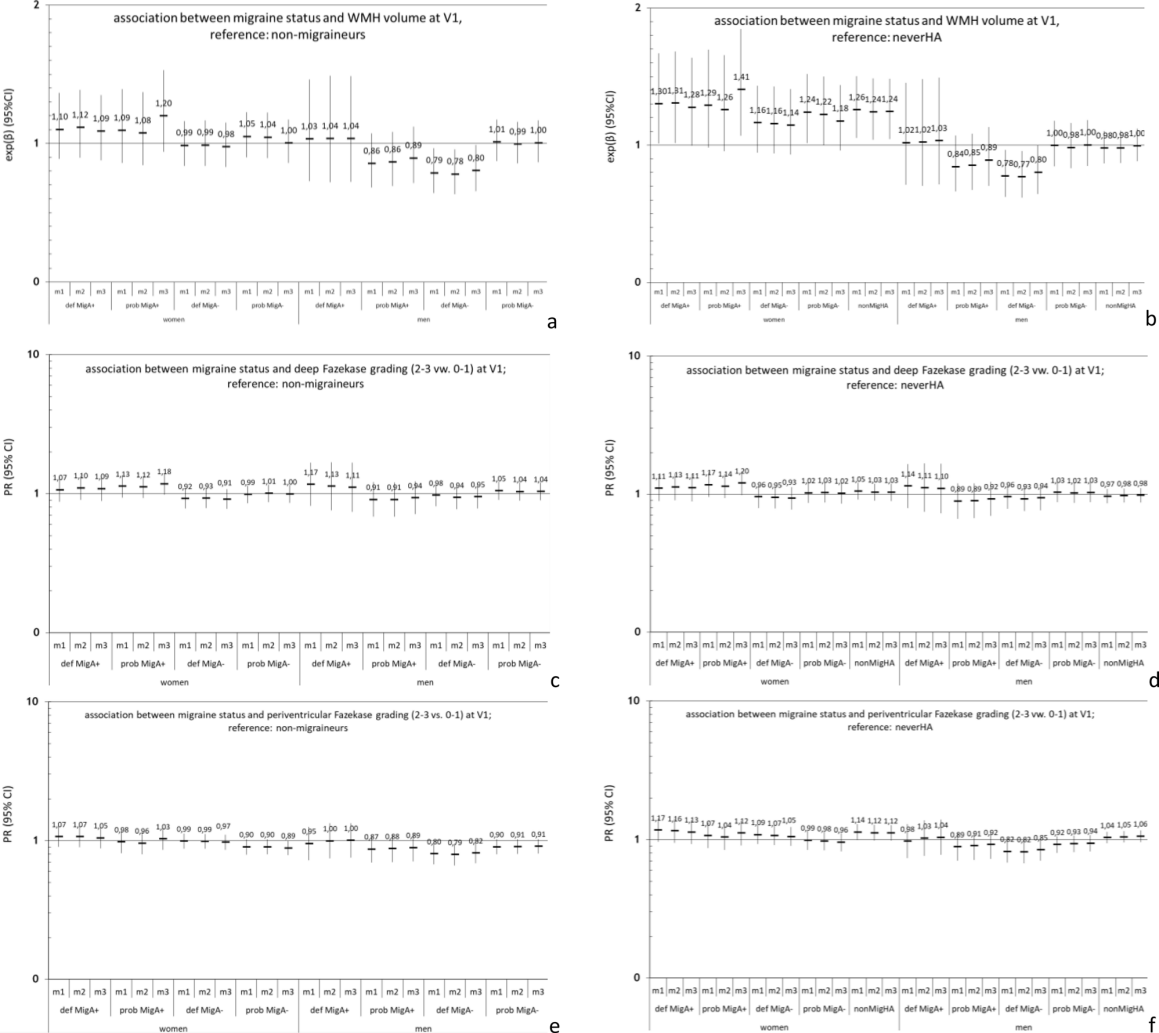
Cross sectional association between headache status and WMH at V1, results of the linear and log- binomial regression; reference a, c,e: participants without migraine; reference b, d,f: neverHA. (**a**, **b**) WMH volume; (**c**, **d**) deep WMH, Fazekas grading 2–3 vs. 0–1; (**e**, **f**) periventricular WMH, Fazekas grading 2–3 vs. 0–1 *MigA+, migraine with aura; MigA-, migraine without aura; neverHA, never headaches; nonMigHA, non-migraine headache; V1, visit 1; PR, prevalence ratio; 95%CI, 95%-confidence interval; m, m1: age adjusted; m2: adjusted as m1 + smoking status (never/past/current), BMI, sport (yes/no), years of education; m3: adjusted as m2 + diabetes melitus, systolic blood pressure, and cholesterol*

Compared to participants without migraine there was no indication in women and men with migraine and its subtypes of having more WMH volume (Fig. 3a). But, women with any headaches (all headache types combined) had a 1.23 [1.04; 1.45]-fold higher WMH volume than women who never had headache. Men with any headaches did not have higher WMH volume than men who never had headache (Fig. 3b). There was no indication of worse deep or periventricular Fazekas grading in women and men with migraine compared to participants without migraine or participants without headache (Fig. 3c-f). High Fazekas grading was very rare in women ≤ 55 years (SM5).

### Descriptive statistics of WMH change

Considering absolute and relative WMH changes over time, women had a higher median and relative WMH progression than men (women: 238 (IQR: -146; 983) mm^3^; men: 109 (IQR: -484; 810) mm^3^; relative change: 7.4% (IQR: -4.1; 20.8) vs. 2.2% (IQR: -15.2; 17.0) (Table 5).

**Table 5:** Characteristics of the female (*n* = 175) and male (*n* = 218) study population at visit 2, stratified by headache status

		Definitive MigA+*	Probable MigA+*	All MigA+**	Definitive MigA-*	Probable MigA-*	All MigA−**	NonMigHA	NeverHA	Total
**women**	**n(%)**	*13 (7.4)*	*13 (7.4)*	26 (14.9)	*36 (20.6)*	*36 (20.6)*	72 (41.1)	54 (30.9)	23 (13.1)	175 (100)
	**WMH vol. diff.** median	*-26.8*	*492*	344	*321*	*221*	229	192	384	238
	**[mm**^**3**^**]** Q1; Q3	*-376; 430*	*-257; 1997*	-27; 826	*-359; 847*	*-177; 1123*	-196; 918	-69; 941	-23; 1221	-146; 983
	**WMH vol. change** median	*-0.8*	*21.2*	10.0	*7.2*	*6.1*	6.1	5.9	8.6	7.4
	**[%]** Q1; Q3	*-9.0; 17.7*	*7.4; 24.9*	-0.8; 22.3	*-13.5; 22.2*	*-7.7; 18.9*	-9.2; 20.5	-2.7; 15.8	-0.4; 30.1	-4.1; 20.8
	**deep Fazekas change**									
	equal or better	*13 (100.0)*	*13 (100.0)*	26 (100.0)	*29 (80.6)*	*33 (91.7)*	62 (86.1)	47 (87.0)	22 (95.7)	157 (89.7)
	worse	*0*	*0*	0	*7 (19.4)*	*3 (8.3)*	10 (13.9)	7 (13.0)	1 (4.3)	18 (10.3)
	**pv Fazekas change**									
	equal or better	*13 (100.0)*	*10 (76.9)*	23 (88.5)	*33 (91.7)*	*33 (91.7)*	66 (91.7)	48 (88.9)	19 (82.6)	156 (89.1)
	worse	*0*	*3 (23.1)*	3 (11.5)	*3 (8.3)*	*3 (8.3)*	6 (8.3)	6 (11.1)	4 (17.4)	19 (10.9)
	**observation [years]**	*3.6 ± 0.5*	*3.3 ± 0.5*	3.4 ± 0.5	*3.6 ± 0.8*	*3.7 ± 0.8*	3.6 ± 0.8	3.7 ± 0.7	3.8 ± 0.7	3.6 ± 0.7
**men**	**n(%)**	*6 (2.8)*	*11 (5.0)*	17 (7.8)	*11 (5.1)*	*26 (11.9)*	37 (17.0)	96 (44.0)	68 (31.2)	218 (100)
	**WMH vol. diff.** median	*84*	*-222*	-131	*284*	*4*	58	40	313	109
	**[mm**^**3**^**]** Q1; Q3	*-388; 1208*	*-637; 119*	-599; 119	*-607; 580*	*-675; 760*	-607; 630	-540; 858	-244; 1166	-484; 810
	**WMH vol. change** median	*2.0*	*-12.5*	-2.2	*5.8*	*-0.9*	1.0	0.7	5.9	2.2
	**[%]** Q1; Q3	*-26.2; 47.4*	*-19.4; 5.3*	-19.4; 5.3	*-22.1; 12.4*	*-26.0; 10.2*	-16.0; 12.4	-16.0; 18.0	-9.9; 18.8	-15.2; 17.0
	**deep Fazekas change**									
	equal or better	*5 (83.3)*	*10 (90.9)*	15 (88.2)	*9 (81.8)*	*23 (88.5)*	32 (86.5)	78 (81.3)	62 (91.2)	187 (85.8)
	worse	*1 (16.7)*	*1 (9.1)*	2 (11.8)	*2 (18.2)*	*3 (11.5)*	5 (13.5)	18 (18.8)	6 (8.8)	31 (14.2)
	**pv Fazekas change**									
	equal or better	*6 (100.0)*	*10 (90.9)*	16 (94.1)	*11 (100.0)*	*21 (80.8)*	32 (86.5)	87 (90.6)	56 (82.4)	191 (87.6)
	worse	*0*	*1 (3.7)*	1 (5.9)	*0*	*5 (19.2)*	5 (13.5)	9 (9.4)	12 (17.6)	23 (12.4)
	**observation [years]**	*3.8 ± 0.5*	*3.2 ± 0.6*	3.4 ± 0.6	*3.6 ± 1.0*	*3.9 ± 0.7*	3.8 ± 0.8	3.7 ± 0.7	3.8 ± 0.7	3.7 ± 0.7

MigA+, migraine with aura; MigA-, migraine without aura; neverHA, never headaches; nonMigHA, non-migraine headache; pv, periventricular; Q1, lower quartile; Q3, upper quartile; vol., volume; WMH, white matter hyperintensity. *definite and probable migraine with and without aura according to modified ICHD-II classification. **definite and probable MigA + and MigA-, respectively, combined

Tables SM6 and SM7 show further characteristics of the study population at V2. The number of participants with Fazekas-score worsening was very low in several migraine subgroups and the percentage in participants with migraine was not increased compared to the other groups (Table 5).

### Association between headache status and WMH change (Fig. 4)

**Fig. 4:**
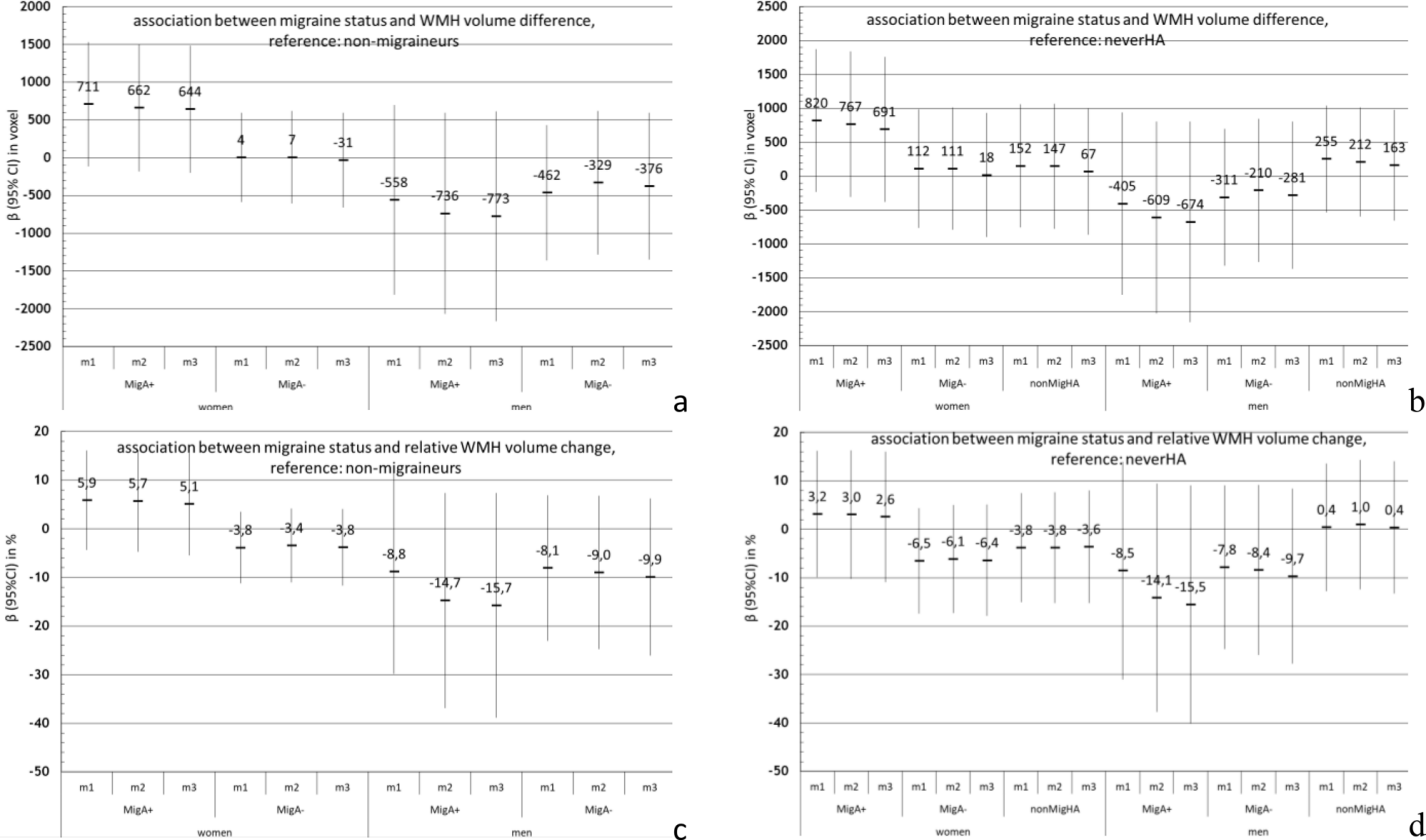
(**a** and **b**) sex-specific association between headache status and WMH volume difference (V2-V1) in mm^3^, (**c** and **d**) sex-specific association between headache status and relative WMH-volume change (V2-V1/V1) in %; reference **a** and **c**: participants without migraine; reference **b** and **d**: neverHA; results of the linear regression. *MigA+, migraine with aura; MigA-, migraine without aura; neverHA, never headaches; nonMigHA, non-migraine headache; V1, visit 1; V2, visit 2; 95%CI, 95% confidence interval; m, model; m1: age adjusted; m2: adjusted as m1 + smoking status (never/past/current), BMI, sport (yes/no), years of education; m3: adjusted as m2 + diabetes melitus, systolic blood pressure, and cholesterol*

Due to the small number, we do not report stratified by definite and possible migraine. In participants with migraine or any headaches there was no evidence of a greater WMH progression (Fig. 4a-d). In fact, we observed a rather negative trend in men with migraine.

## Discussion

We examined among women and men whether participants with MigA + and MigA- or any headaches show more WMH at study baseline and more WMH progression during follow-up than participants without migraine or participants who stated that they never had headaches. Combining all headache types, women with any headaches had a 1.23 [1.04; 1.45]-fold higher WMH volume than women who never had headaches. Compared to women without migraine (nonMigHA and neverHA combined), there was no evidence of greater WMH volume in women with migraine with and without aura. There was no evidence of greater WMH progression in women with migraine or any headaches compared to women without migraine or women who never had headache. In men, there was no evidence of a higher amount of WMH or progression in men with migraine or any headaches. Indeed, even negative trends were observed in men with migraine. High WMH volumes and high Fazekas scores were uncommon in participants ≤ 55 years. For this reason, and because there were fewer young participants in our cohort, our measured effects were based predominantly on data for those over 55 years. Thus, in our study, there was some indication that WMH were more pronounced in older women with any headaches including migraine, but not in men.

The cross-sectional and longitudinal association between migraine or any headaches and WMH has been analyzed previously, but the definitions of headache status, reference group, and the measurement of WMH differ substantially across studies, and the results differ. From 1993 to 1995, *n* = 1949 participants of the Atherosclerosis Risk in Communities cohort study received MRI. WMH severity was graded according to the Cardiovascular Health Study grading system with 0 to 9 scale. MigA- was associated with an increased risk of severe white matter disease (score of ≥ 3, odds ratio = 1.87 [1.04, 3.37]), whereas MigA + was not (0.55 [0.17; 1.83]). Also, participants with nonMigHA had a weak indication for having more WMH compared to participants without severe headache. From 2004 to 2006, *n* = 1028 participants underwent a second MRI. Images were analyzed also using a semiautomated volumetric analysis. Individuals with migraine had on average of 2.65 [0.06; 5.24] cm^3^ more WMH than those without headache, whereas nonMigHA was not associated with greater WMH volume (− 0.77 [− 3.54; 2.10] cm^3^) [11]. Among women of a population-based study with Dutch adults, the chance for high deep WMH load (≥ 80. percentile vs. ≤ 20. percentile) was increased in patients with migraine compared with controls (no severe headaches) (OR: 2.1 [1.0; 4.1]). The chance increased with attack frequency and was similar in patients with MigA + or MigA-. Men with migraine and controls did not differ in the presence of WMH and there was no association between severity of periventricular WMH and migraine [29]. Another population-based study found an association between any history of severe headache and increasing volume of WMH. The adjusted OR of being in the highest third for total volume of WMH was 2.0 [1.3; 3.1] for participants with any history of severe headache when compared with participants without severe headache. The association pattern was similar for all headache types [15]. A meta-analysis demonstrated that individuals with migraine with aura had a 1.68 [1.07; 2.65]-fold higher and individuals with migraine without aura a 1.34 [0.96; 1.87]-fold higher chance of having WMH than controls [10]. A study with twins from the Danish Twin Registry reported no higher Fazekas or Scheltens’ scores in individuals with migraine with aura compared to controls without migraine, but there was a weak indication of individuals with migraine with aura having slightly higher total WMH volume compared to unrelated controls (mean difference: 0.17 [-0.08; 0.41] cm^3^) and in twin pairs discordant for migraine with aura (0.21 [-0.20; 0.63] cm^3^) [30]. Another population-based study reported that participants with tension-type headache were more likely to have extensive WMH (Fazekas scale ≥ 2) than headache-free participants (OR: 2.46 [1.44; 4.20], but not those with migraine or unclassified headache. Those with new onset headache were more likely to have WMH than those who were stable headache-free (OR: 2.24 [1.13–4.44]) [31]. The population-based Northern Manhattan Study reported no association between migraine with or without aura and WMH volume [32]. Concerning the longitudinal change in WMH, in the above mentioned Atherosclerosis Risk in Communities cohort study there was a weak indication for individuals with migraine having more WMH-volume progression compared to those without migraine (1.58 [-0.37, 3.53] cm^3^) over a study period of 8–12 years [11]. Another population-based study reported that in women with migraine, the proportion of increased deep WMH after 9 years (∆ between follow-up WMH-volume and baseline ≥ 0.01 ml) was higher than in those without migraine (OR: 2.1; [1.0; 4.1]). No association between attack frequency and WMH progression was found and no association was observed in men [12]. Two longitudinal clinical studies with small case numbers and no control groups found an association between aura duration as well as number of migraine attacks and number of new lesions [14] and an increase of the WMH size [13]. Although previous study results are heterogeneous and contradictory, it seems that headache itself and the severity of the headache are more associated with the extent of WMH rather than the type of headache. In addition to our study, others have also reproduced, that this seems to be more pronounced in women [12, 29]. It can be assumed that the very heterogeneous choice of controls strongly influence the results, as well as the different gender distribution within study groups of individuals with migraine.

In the context of the above discussion, there is an important aspect to consider. The studies listed above enrolled individuals with migraine at a time when migraine was active. Associations between migraine and cerebrovascular risk have also been demonstrated when migraine was active [33]. In our study, however, any headache history was considered without distinguishing between current and past headaches or headache frequency at the time of interview. This study design clearly distinguishes our study from previous ones.

In our study there were many participants whose Fazekas score improved within 3.7 ± 0.7 years. Lesion remission was reported before [13, 34]. Remission was more likely in small WMH and patients with low attack frequency [13]. WMH decrease could be related to the fact that signal changes in FLAIR or T2-weighted MRI sequences are to a large extent dependent on fluid shifts and not only a representation of permanent myelin or axonal damage [9]. Thus, portions of lesions could be only temporary changes that do not show up after some time due to resorption processes and new fluid shifts. This volatility of WMH might be an explanation for the heterogeneous study results mentioned before.

It was hypothesized that the association between migraine and WMH or its progression may be more due to changes occurring at a younger age and that these early changes would be difficult to detect in older age due to the accumulation of other risk factors, i.e. cardiovascular risk factors [11, 12]. In our cohort, the proportion of younger participants was too small and the occurrence of larger WMH in those uncommon that we could not investigate this any further.

WMH in MRI scans are a common sign of aging, but they can also indicate mild damage. Increased MRI signal intensity in the white matter can be caused by various vascular and non-vascular pathologies [35, 36]. In clinical MRI scans of older individuals, WMHs are often interpreted as a surrogate for cerebral small vessel disease [35, 37, 38]. However, distinguishing WMHs caused by small vessel disease from those caused by multiple sclerosis and other inflammatory brain diseases, or metabolic leukodystrophies can be challenging [39]. Additionally, cortical degeneration, which is common in older individuals with degenerative diseases like Alzheimer’s disease, can result in the degeneration of fiber tracts and subsequent MRI changes [39]. Therefore, WMHs are not specific enough for diagnosis.

Currently, the clinical and functional significance of WMH is uncertain. However, WMH in individuals with migraine are often detected on imaging as an incidental finding in the evaluation of secondary headache, which may be a cause for concern for both the neurologist and the patient [40]. However, whether sufficient headache prevention and therapy has a positive effect on the development of WMH could be included in future prospective headache studies with imaging.

## Strengths and limitations

One strength of our study is the high data quality from the 1000BRAINS study. Another strength is that we had quantitative (volume) and qualitative (Fazekas classification) WMH data. To maximize blinding of our independent raters, no comparison of V1 and V2 was performed. In case of different initial classifications, consensus was reached. Limitations are the possible migraine misclassifications, because correctly diagnosing migraine is challenging and ideally requires a clinical interview conducted by a headache specialist. Also, accurately diagnosing MigA + was crucial in our context because patients with other neurological conditions involving transient neurological symptoms, particularly transient ischemic attacks (TIAs), may be mistakenly diagnosed with MigA+. Since patients with TIAs have a higher burden of WMH, this type of misdiagnosis could result in a false association between MigA + and WMH. Because MigA + without visual disturbances is rare and a condition with weakness in the arm/leg carries a higher risk of misdiagnosed TIA [41, 42]), we excluded all participants with weakness in the arm/leg immediately before the headache and all participants without visual disturbance symptoms but with tingling/deafness or speech disorders immediately before the headache and performed a sensitivity analysis; the estimates remained similar (data not shown). Further limitations are that the headache questionnaire was administered only once before first MRI, it was collected whether various migraine symptoms ever occurred in life, and the questionnaire was not validated specifically for diagnosing MigA+. Because the majority of older participants probably no longer had active migraine at the time of the survey due to advanced age, it is also conceivable that they reported fewer classic migraine symptoms from the past. Therefore, an underestimation of the lifetime prevalence of migraine cannot be ruled out. Despite using the IHS criteria [16] misclassification was most likely present due to this approach.

To quantify possible misclassification we performed a quantitative bias analysis (SM8). In women, the estimated true lifetime prevalence migraine (definite and probable migraine with and without aura combined) was unchanged from that measured (50.6% vs. 50.4%), in men it was lower (16.8% vs. 26.8%). Prevalences appear to be very high. However, analyses with persons with definite and probable migraine yielded similar results. Another limitations is that we do not have information about the severity of migraine and nonMigHA.

## Conclusion and clinical implications

Our study demonstrated no increased occurrence or progression of WMH in participants with migraine. Although study results to date are inconsistent and contradictory, the headache itself and the severity of the headache are probably more related to the extent of WMH than the type of headache, and this appears to be more pronounced in women. Our study also provides some indication for a higher WMH volume in women with any headaches including migraine. The underlying pathomechanisms and why this association was not shown in our male study population remain unclear and require further research. In particular, the question of previously unknown influencing factors in adolescence or young adulthood and hormonal factors in women are of great interest. Studies with large collectives including all headache subtypes, long follow-up, starting at young age, detailed data on the use of birth control pills, hormone replacement therapy and menopause in women, and more data about headache frequency and attack duration are needed to investigate the course of WMH in migraine and headache in detail. It would also be useful to record the location of the WMH even more precisely. Subcortical WMH should be recorded and a subdivision by cerebral lobe and side should also be made. Whether sufficient headache prevention and treatment have protective effects on WMH should also be included in future prospective headache studies with imaging.

### Electronic supplementary material

Below is the link to the electronic supplementary material.

Supplementary Material 1

## Data Availability

The corresponding author has full access to all data in the study. Due to data security reasons (i.e., data contain potentially participant identifying information), the 1000BRAINS study does not allow sharing data as a public use file. Data requests can be addressed to recall@uk-essen.de.

## Abbreviations

95%CI: 95%-confidence intervals
β: beta
BIANCA: Brain Intensity AbNormality Classification Algorithm
BMI: body mass index
DAG: directed acyclic graph
FLAIR: fluid-attenuated inversion recovery
HDL: high-density lipoprotein
HNR: Heinz Nixdorf Recall
ICHD-II: international classification of headache disorders, 2nd edition
HIS: International Headache Society
IQR: inter quartile range
ISCED-97: International Standard Classification of Education
LDL: low-density lipoprotein
M: model
MGS: Heinz Nixdorf Multigeneration Study
MigA+: migraine with aura
MigA-: migraine without aura
MRI: magnetic resonance imaging
neverHA: never headache
nonMigHA: non-migraine headache
PR: prevalence ratios
PV: periventricular
Q: quartile
SD: standard deviation
SM: supplementary material
TE: echo time
TIA: transient ischemic attack
TR: repetition time
V1: visit 1
V2: visit 2
WMH: white matter hyperintensities
