# Supplementary material for: Migraine or any headaches and white matter hyperintensities and their progression in women and men
Source: J Headache Pain. 2024 May 15;25(1):78. doi: 10.1186/s10194-024-01782-7 (PMC11094904; doi:10.1186/s10194-024-01782-7)
Supplement: Supplementary file 1 — Supplementary Material 1 [file 10194_2024_1782_MOESM1_ESM.docx]

**Supplementary material (SM)**

**SM1.** Information on the Heinz Nixdorf Recall (HNR) study, the Heinz Nixdorf Multigeneration Study (MGS) and the 1000BRAINS study

Heinz Nixdorf Recall (HNR) study

Detailed information on the HNR study was published previously [Schmermund et al., 2002, Am Heart J 144, 212-218]. Briefly, the ongoing population-based prospective HNR study collects data on health, social and environmental risk factors and cardiovascular outcomes in the Ruhr metropolitan region. The study population is composed of random samples from the population registers of the cities of Bochum, Essen, and Mülheim/Ruhr. Between 2000 and 2003, 4814 participants (aged 45-75 years, 50.2% women) were included in the baseline survey, with a recruitment rate of 55.8% [Stang et al., 2005, Eur J Epidemiol 20, 489-496]. The study was approved by the ethics committee of the University of Duisburg-Essen, all participants gave their written consent to conduct the study, and the study is certified and recertified according to DIN ISO 9001:2000/2008. Five- and 10-year follow-up examinations were performed, and participants still receive annual health questionnaires.

Heinz Nixdorf Multigeneration Study (MGS)

The MGS is an extension of the HNR study. From 2013-2016, the partners and adult children of HNR subjects aged 18-90 years were included in the study for the first time. The aim is to investigate cardiovascular risk factors and the incidence of cardiovascular disease in more detail, particularly in families. The study protocol and examination program of the first survey of the MGS is largely similar to that of the 10-year follow-up of the HNR study. Subjects are followed-up with annual health questionnaires [Kowall et al., 2021, PLoS One 16, e0252828]. In 2020, first follow-up of the MGS began.

1000BRAINS study

Detailed information on the 1000BRAINS study was published previously [Caspers et al., Front Aging Neurosci 2014, 6, 149]. Briefly, the 1000BRAINS study is a project initiated by the Institute of Neuroscience and Medicine of Forschungszentrum Jülich in cooperation with the University of Duisburg-Essen. It aims to investigate structural and functional brain differences during the natural aging process. The 1000BRAINS study participants were recruited exclusively from the already existing cohorts of the HNR study and the MGS. Participants were excluded from participating if they fulfilled the exclusion criteria of the 1000BRAINS study which were guided by ethical standards and safety guidelines regarding the use of MRI in healthy subjects for research purposes only. According to those guidelines, persons with the following contraindications did not receive MRI: coronary artery stents, pacemakers, surgical implants or prostheses in the trunk or head, claustrophobia, history of neurosurgical procedures, presence of tattoos or permanent makeup on the head. Relative contraindications were dental implants and dental bridges. Examinations with artifacts were canceled. MRI examinations were performed on a 3-T MR scanner (Tim-TRIO, Siemens Medical Systems) at the Jülich Research Center, Germany. N=1254 MRI scans of the skull were obtained at the first visit (V1) from 2011-2014 and n=438 after 3.7±0.7 years (second visit, V2). Different sequences were applied: an anatomical 3D-T1 weighted MPRAGE sequence, a 3D-T2 weighted SPACE sequence, MR angiography of the basal cerebral arteries without contrast using a time-of-flight (ToF) sequence, different diffusion-weighted sequences, and a clinical T2-weighted FLAIR sequence.


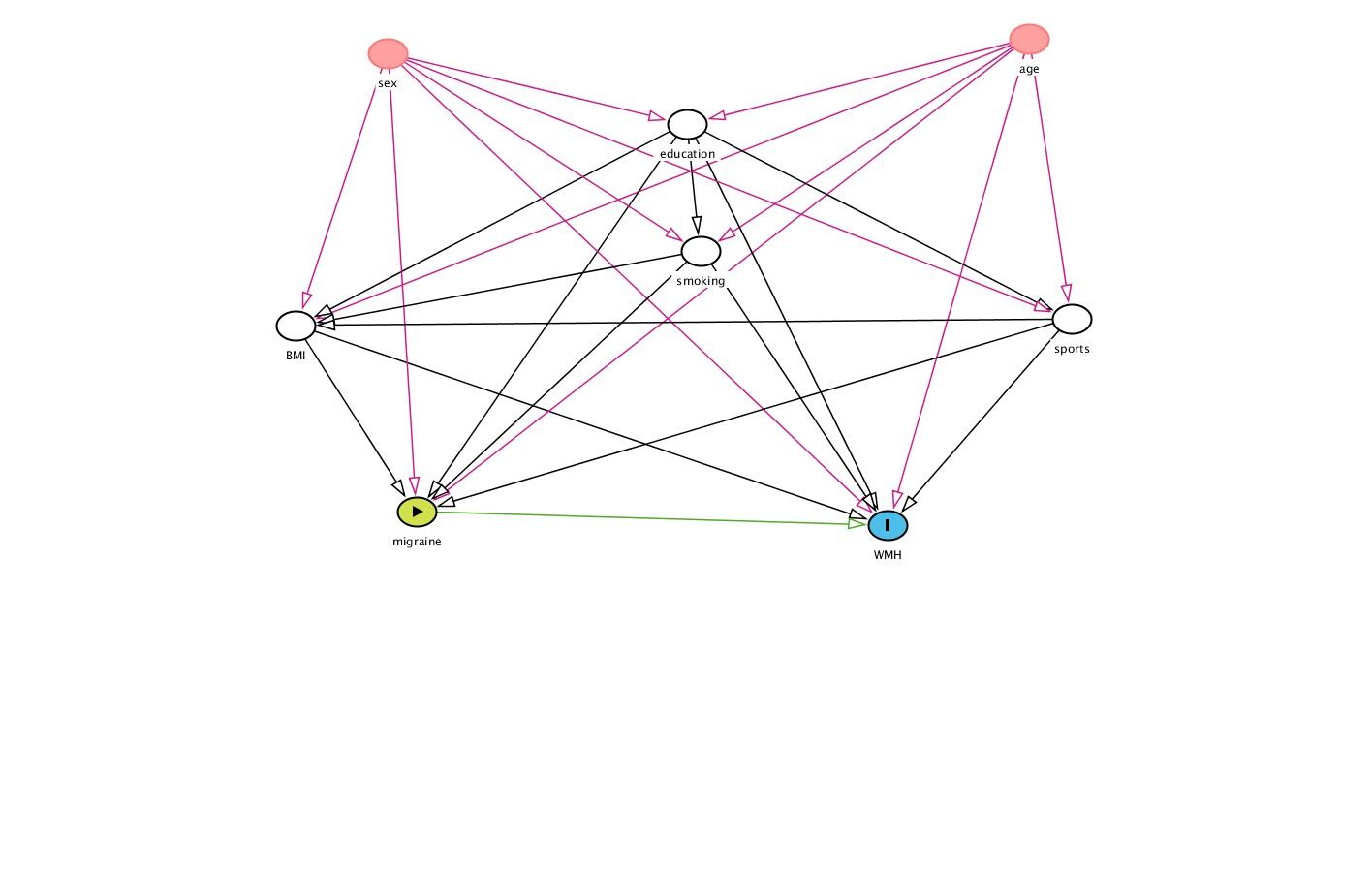


**SM2.** Directed acyclic graph (DAG, Textor J, et al. International Journal of Epidemiology. 2016;45(6):1887-94]) used to determine the minimal sufficient adjustment set that includes the confounding factors of BMI, education, smoking and sports exercise to estimate the sex-stratified effect of migraine status on WMH**.**

**SM3.** Age distribution in the female and male study population at visit 1 (V1) in n(%).

|  |  | **MigA+** | **MigA−** | **nonMigHA** | **neverHA** | **total** |
| --- | --- | --- | --- | --- | --- | --- |
| **women** | **n(%)** | 70 (14.6) | 172 (35.8) | 169 (35.2) | 69 (14.4) | 480 (100) |
|  | **≤ 55 years** | 18 (17.5) | 50 (48.5) | 31 (30.1) | 4 (3.9) | 103 (21.5) |
|  | **>55 years** | 52 (6.6) | 122 (32.4) | 138 (36.6) | 65 (17.2) | 377 (78.5) |
| **men** | **n(%)** | 42 (7.2) | 114 (19.6) | 257 (44.2) | 169 (29.0) | 582 (100) |
|  | **≤ 55 years** | 17 (12.9) | 31 (23.5) | 65 (49.2) | 19 (14.4) | 132 (22.7) |
|  | **>55 years** | 25 (5.6) | 83 (18.4) | 192 (42.7) | 150 (33.3) | 450 (77.3) |

**SM4:** Scatter plot of age and WMH volume at V1.

***Legend:*** *WMH, white matter hyperintensities; V1, visit 1.*

**SM5.** Fazekas grading at V1, stratified by headache status, sex and age group.

|  | | MigA+ | | MigA- | | nonMigHA | | neverHA | | total | | MigA+ | | MigA- | | nonMigHA | | neverHA | | total | |
| --- | --- | --- | --- | --- | --- | --- | --- | --- | --- | --- | --- | --- | --- | --- | --- | --- | --- | --- | --- | --- | --- |
|  |  | N | % | N | % | N | % | N | % | N | % | N | % | N | % | N | % | N | % | N | % |
|  | | women, ≤55 years | | | | | | | | | | women, >55 years | | | | | | | | | |
| grade | 0 | 4 | 22.2 | 9 | 18.0 | 5 | 16.1 | 1 | 25.0 | 19 | 18.4 | . | . | 3 | 2.5 | . | . | . | . | 3 | 0.8 |
|  | 1 | 13 | 72.2 | 35 | 70.0 | 20 | 64.5 | 3 | 75.0 | 71 | 68.9 | 18 | 34.6 | 54 | 44.3 | 55 | 39.9 | 25 | 38.5 | 152 | 40.3 |
|  | 2 | . | . | 5 | 10.0 | 6 | 19.4 | . | . | 11 | 10.7 | 26 | 50.0 | 46 | 37.7 | 53 | 38.4 | 29 | 44.6 | 154 | 40.8 |
|  | 3 | 1 | 5.6 | 1 | 2.0 | . | . | . | . | 2 | 1.9 | 8 | 15.4 | 19 | 15.6 | 30 | 21.7 | 11 | 16.9 | 68 | 18.0 |
|  | | men, ≤55 years | | | | | | | | | | men, >55 years | | | | | | | | | |
| grade | 0 | 5 | 29.4 | 7 | 22.6 | 10 | 15.4 | 4 | 21.1 | 26 | 19.7 | 1 | 4.0 | 1 | 1.2 | 1 | 0.5 | 2 | 1.3 | 5 | 1.1 |
|  | 1 | 10 | 58.8 | 20 | 64.5 | 38 | 58.5 | 12 | 63.2 | 80 | 60.6 | 10 | 40.0 | 38 | 45.8 | 85 | 44.3 | 56 | 37.3 | 189 | 42.0 |
|  | 2 | 2 | 11.8 | 4 | 12.9 | 17 | 26.2 | 3 | 15.8 | 26 | 19.7 | 11 | 44.0 | 37 | 44.6 | 74 | 38.5 | 59 | 39.3 | 181 | 40.2 |
|  | 3 |  |  |  |  |  |  |  |  |  |  | 3 | 12.0 | 7 | 8.4 | 32 | 16.7 | 33 | 22.0 | 75 | 16.7 |

***Legend:*** *MigA+, migraine with aura; MigA-, migraine without aura; neverHA, never headaches; nonMigHA, non-migraine headache.*

**SM6.** Characteristics of the female (n=175) study population at V2, stratified by headache status; n(%) and mean±SD.

|  | | **MigA+** | **MigA−** | **nonMigHA** | **neverHA** | **total** |
| --- | --- | --- | --- | --- | --- | --- |
| **n(%)** | | 26 (14.9) | 72 (41.1) | 54 (30.9) | 23 (13.1) | 175 (100) |
| **‚definitive‘ migraine** | | 13 (50.0) | 36 (50.0) | - | - | - |
| **age [years]** | | 58.0+13.1 | 60.9+11.7 | 60.6+9.6 | 65.7+6.5 | 61.0+10.9 |
| **≤ 55 years** | | 8 (30.8) | 14 (19.4) | 11 (20.4) | 0 | 33 (18.9) |
| **>55 years** | | 18 (69.2) | 58 (80.6) | 43 (79.6) | 23 (100) | 142 (81.1) |
| **smoking** | never | 13 (56.5) | 33 (45.8) | 30 (52.6) | 12 (52.2) | 88 (50.3) |
|  | past | 5 (21.7) | 30 (41.7) | 20 (35.1) | 9 (39.1) | 64 (36.6) |
|  | current | 5 (21.7) | 9 (12.5) | 7 (12.3) | 2 (8.7) | 23 (13.1) |
| **BMI [kg/m²]** | | 25.5+4.1 | 27.6+4.9 | 26.9+5.6 | 26.8+4.6 | 27.0+5.0 |
| missing | |  | 1 |  |  | 1 |
| **sport** | yes | 16 (69.6) | 43 (59.7) | 42 (73.7) | 17 (73.9) | 118 (67.4) |
|  | no | 7 (30.4) | 29 (40.3) | 15 (26.3) | 6 (26.1) | 57 (32.6) |
| **education [years]** | | 14.7+2.4 | 14.1+2.3 | 14.5+2.4 | 14.3+2.3 | 14.4+2.3 |
| missing | |  | 1 |  |  | 1 |
| **systolic RR [mmHg]** | | 119.9+14.3 | 120.0+16.7 | 119.8+14.7 | 119.0+12.5 | 119.8+15.1 |
| **diabetes mellitus** | yes | 1 (4.3) | 9 (12.5) | 3 (5.3) | 2 (8.7) | 15 (8.6) |
|  | no | 22 (95.7) | 63 (87.5) | 54 (94.7) | 21 (91.3) | 160 (91.4) |
| **total cholesterol [mg/dl]** | | 210.1+38.9 | 232.9+40.4 | 224.8+37.0 | 216.8+36.9 | 225.1+39.2 |
| missing | |  | 1 | 1 |  | 2 |
| **LDL cholesterol [mg/dl]** | | 118.4+30.7 | 140.8+35.3 | 131.4+27.0 | 125.4+31.3 | 132.7+32.3 |
| missing | |  | 1 |  |  | 1 |
| **HDL cholesterol [mg/dl]** | | 70.5+16.6 | 70.7+18.0 | 72.1+16.6 | 70.0+18.3 | 71.0+17.3 |
| missing | |  | 1 |  |  | 1 |
| **WMH vol. V1 [mm^3^]** | |  |  |  |  |  |
| median | | 4103 | 4159 | 4580 | 3829 | 4144 |
| Q1; Q3 | | 2066; 6805 | 2517; 6868 | 2946; 7504 | 2596; 4456 | 2485; 6805 |
| **WMH vol. V2 [mm^3^]** | |  |  |  |  |  |
| median | | 3959 | 4476 | 4873 | 3836 | 4317 |
| Q1; Q3 | | 2450; 7312 | 2187; 7425 | 3130; 7903 | 2815; 5592 | 2615; 7516 |

***Legend:*** *BMI, body mass index; LDL, low-density lipoprotein; HDL,* *high-density lipoprotein; MigA+, migraine with aura; MigA-, migraine without aura; neverHA, never headaches; nonMigHA, non-migraine headache; Q1, lower quartile; Q2, median; Q3, upper quartile; RR, blood pressure; SD, standard deviation; vol., volume; WMH, white matter hyperintensity; V1, visit 1; V2, visit 2. *‘probable and definitive migraine with and without aura, respectively’ and **’definitive migraine’ according to modified ICHD-II classification.*

**SM7.** Characteristics of the male (n=218) study population with WMH measurement at V2, stratified by migraine status; n(%) and mean±SD.

|  | | **MigA+** | **MigA−** | **nonMigHA** | **neverHA** | **total** |
| --- | --- | --- | --- | --- | --- | --- |
| **n(%)** | | 17 (7.8) | 37 (17.0) | 96 (44.0) | 68 (31.2) | 218 (100) |
| **‚definitive‘ migraine** | | 6 (35.3) | 11 (29.7) | - | - | - |
| **age [years]** | | 58.1+13.3 | 60.1+10.5 | 59.7+13.2 | 63.3+11.3 | 60.7+12.3 |
| **≤ 55 years** | | 6 (35.3) | 6 (16.2) | 24 (25.0) | 10 (14.7) | 46 (21.1) |
| **>55 years** | | 11 (64.7) | 31 (83.8) | 72 (75.0) | 58 (85.3) | 172 (78.9) |
| **smoking** | never | 5 (33.3) | 13 (35.1) | 44 (44.9) | 27 (39.7) | 89 (40.8) |
|  | past | 8 (53.3) | 21 (56.8) | 43 (43.9) | 35 (51.5) | 107 (49.1) |
|  | current | 1 (6.7) | 3 (8.1) | 11 (11.2) | 6 (8.8) | 21 (9.6) |
| missing | | 1 |  |  |  | 1 |
| **BMI [kg/m²]** | | 27.3+2.2 | 29.1+4.8 | 27.4+3.7 | 27.5+3.7 | 27.7+3.9 |
| missing | |  | 1 | 1 |  | 2 |
| **sport** | yes | 12 (80.0) | 30 (81.1) | 65 (66.3) | 47 (69.1) | 154 (70.6) |
|  | no | 2 (13.3) | 7 (18.9) | 33 (33.7) | 21 (30.9) | 63 (28.9) |
| missing | | 1 |  |  |  | 1 |
| **education [years]** | | 14.7+2.5 | 16.5+1.7 | 15.5+2.1 | 15.9+2.1 | 15.8+2.1 |
| **systolic RR [mmHg]** | | 132.3+16.7 | 127.4+16.7 | 129.8+16.2 | 135.2+16.3 | 131.2+16.5 |
| missing | | 1 |  |  |  | 1 |
| **diabetes mellitus** | yes | 2 (13.3) | 4 (10.8) | 13 (13.3) | 16 (23.5) | 35 (16.1) |
|  | no | 13 (86.7) | 33 (89.2) | 85 (86.7) | 52 (76.5) | 183 (83.9) |
| **total cholesterol [mg/dl]** | | 214.5+39.5 | 198.5+26.4 | 204.4+44.0 | 209.5+34.4 | 205.6+38.3 |
| missing | | 1 |  |  |  | 1 |
| **LDL cholesterol [mg/dl]** | | 135.0+39.0 | 121.4+28.1 | 127.4+36.1 | 126.9+34.6 | 126.7+34.5 |
| missing | | 1 |  |  |  | 1 |
| **HDL cholesterol [mg/dl]** | | 55.5+18.0 | 50.2+8.5 | 57.0+15.0 | 58.1+16.4 | 56.1+15.0 |
| missing | | 1 |  |  |  | 1 |
| **WMH vol. V1 [mm^3^]** | |  |  |  |  |  |
| median | | 4209 | 4662 | 4472 | 5071 | 4718 |
| Q1; Q3 | | 1816; 5807 | 3184; 5841 | 2823; 6713 | 3154; 10010 | 2919; 7516 |
| **WMH vol. V2 [mm^3^]** | |  |  |  |  |  |
| median | | 4570 | 4454 | 4322 | 5017 | 4637 |
| Q1; Q3 | | 1778; 5979 | 2658; 6305 | 2841; 7396 | 3005; 9238 | 2750; 7681 |

***Legend:*** *BMI, body mass index; LDL, low-density lipoprotein; HDL,* *high-density lipoprotein; MigA+, migraine with aura; MigA-, migraine without aura; neverHA, never headaches; nonMigHA, non-migraine headache; Q1, lower quartile; Q2, median; Q3, upper quartile; RR, blood pressure; SD, standard deviation; vol., volume; WMH, white matter hyperintensity; V1, visit 1; V2, visit 2. *‘probable and definitive migraine with and without aura, respectively’ and **’definitive migraine’ according to modified ICHD-II classification.*

**SM8. Bias analysis: Possible misclassification of migraine**

To estimate the true prevalence of migraine in our study cohort we performed a bias analysis. We used the diagnostic indices (sensitivity: 85% and specificity: 85%) for migraine obtained from the validation study [Yoon MS, et al. Cephalalgia 2008; 605–608]. If A* is the number of participants classified with migraine, N is the total number of individuals, Se is the sensitivity and Sp is the specificity, the number of true migraine subjects (A) is calculated as:

A = A* - (1 - Sp) • N) / (Se + Sp – 1) [Fox MP, MacLehose RF, Lash TL. Applying quantitative bias analysis to epidemiologic data. 2nd ed. Cham: Springer; 2021].

For possible and definitive migraine with and without aura in women:

A*=242; Fp=0.15; N=480; Se=0.85; Sp=0.85

A = (242 - 0.15 • 480) / (0.85 + 0.85 - 1) = 242.86

estimated true prevalence: 243/480=50.6%

For possible and definitive migraine with and without aura in men:

A*=156; Fp=0.15; N=582; Se=0.85; Sp=0.85

A = (156 - 0.15 • 582) / (0.85 + 0.85 - 1) = 98.14

estimated true prevalence: 98/582=16.8%

In women, the estimated true prevalence of migraine cases is unchanged from that measured (50.6% vs. 50.4%); in men, the estimated true prevalence of migraine cases is lower (16.8% vs. 26.8%).

**SM9. Detailed acknowledgements and funding of the HNR study, MGS, and 1000BRAINS**

Detailed acknowledgments

The authors express their gratitude to all study participants of the Heinz Nixdorf Recall (HNR) study, the personnel of the HNR study center and the EBT-scanner facilities, the investigative group and all former employees of the HNR study. The authors thank the 1000BRAINS personnel at the Research Centre Jülich. The authors also thank the Advisory Board of the HNR Study: T. Meinertz, Hamburg, Germany (Chair); C. Bode, Freiburg, Germany; P.J. de Feyter, Rotterdam, Netherlands; B. Güntert, Hall i.T., Austria; F. Gutzwiller, Bern, Switzerland; H. Heinen, Bonn, Germany; O. Hess (†), Bern, Switzerland; B. Klein (†), Essen, Germany; H. Löwel, Neuherberg, Germany; M. Reiser, Munich, Germany; G. Schmidt (†), Essen, Germany; M. Schwaiger, Munich, Germany; C. Steinmüller, Bonn, Germany; T. Theorell, Stockholm, Sweden; and S.N Willich, Berlin, Germany.

Detailed funding

The authors thank the Heinz Nixdorf Foundation [Chairman: Martin Nixdorf; Past Chairman: Dr jur. Gerhard Schmidt (†)], for their generous support of this study. Parts of the study were also supported by the German Research Council (DFG) [DFG project: EI 969/2-3, ER 155/6-1;6-2, HO 3314/2-1;2-2;2-3;4-3, INST 58219/32-1, JO 170/8-1, KN 885/3-1, PE 2309/2-1, SI 236/8-1;9-1;10-1,], the German Ministry of Education and Science [BMBF project: 01EG0401, 01GI0856, 01GI0860, 01GS0820_WB2-C, 01ER1001D, 01GI0205], the Ministry of Innovation, Science, Research and Technology, North Rhine-Westphalia (MIWFT-NRW), the Else Kröner-Fresenius-Stiftung [project: 2015_A119], the German Social Accident Insurance [DGUV project: FF-FP295], and the German Heart Foundation. Furthermore the study was supported by the Competence Network for HIV/AIDS, the deanship of the University Hospital and IFORES of the University Duisburg-Essen, the European Union, the German Competence Network Heart Failure, Kulturstiftung Essen, the Protein Research Unit within Europe (PURE), the Dr. Werner-Jackstädt Stiftung and the following companies: Celgene GmbH München, Imatron/GE-Imatron, Janssen, Merck KG, Philips, ResMed Foundation, Roche Diagnostics, Sarstedt AG&Co, Siemens HealthCare Diagnostics, Volkswagen Foundation.

The authors thank the Heinz Nixdorf Foundation (Chairman: Martin Nixdorf) for their generous support of the Heinz Nixdorf Recall MultiGeneration Study.

Additionally, 1000BRAINS was funded by European Union’s Horizon 2020 Research and Innovation Programme under grant agreement no. 945539 (HBP SGA3; S.C.) as well as from the Initiative and Networking Fund of the Helmholtz Association (S.C.).

Statement

The corresponding author has full access to all data in the study. Due to data security reasons (i.e., data contain potentially participant identifying information), the HNR study and 1000BRAINS do not allow sharing data as a public use file. Data requests can be addressed to recall@uk-essen.de.
